# Financial adversity and subsequent health and wellbeing during the COVID-19 pandemic in the UK: A qualitative interview study

**DOI:** 10.1016/j.ssmqr.2023.100224

**Published:** 2023-06

**Authors:** Tom May, Henry Aughterson, Daisy Fancourt, Alexandra Burton

**Affiliations:** Research Department of Behavioural Science and Health, Institute of Epidemiology & Health Care, University College London, 1-19 Torrington Place, London, WC1E 7HB, UK

**Keywords:** Financial adversity, Mental health, Wellbeing, COVID-19, Qualitative, Social determinants, Inequalities

## Abstract

**Aims:**

There are concerns that the economic impacts of the COVID-19 pandemic, including employment inactivity and job loss, will have consequences for the UK population's health and wellbeing. However, there is limited qualitative research into how financial adversity contributes to poor health outcomes in this context. This study aimed to explore forms of financial adversity experienced during the pandemic and their subsequent impacts for health and wellbeing.

**Methods:**

Qualitative semi-structured interviews with 20 people who experienced a form of financial adversity during the pandemic and six service providers employed in social welfare support services. Data were analysed using reflexive thematic analysis.

**Results:**

Two main sources of financial adversity were identified: reductions in household incomes and increased living costs which engendered emotional and physical burdens. Coping strategies included increased financial borrowing, support from informal and formal networks and cutting back on energy use, food and non-essential items.

**Conclusion:**

Our study highlighted exposure to multiple financial adversities because of the pandemic and how these experiences led to poor mental and physical health. The findings underline the importance of measures attending to the immediate needs of individuals, including accessible, co-located financial and psychological services, as well as broader measures that seek to reduce social and economic inequalities.

## 1 Introduction

The COVID-19 pandemic and measures imposed to suppress the virus have not only had implications for the mental health and wellbeing of the global population (Brooks et al., 2020) but consequences for the economy and its workforce (International Labour Organization, 2020). Globally, lockdown measures, including self-isolation, physical distancing and the closure of non-essential business settings, have led to forms of financial adversity including job loss and immediate reductions in work hours and income, for many workers worldwide (Hensher, 2020). In March 2020, 1.2 million people in the UK applied for Universal Credit, approximately a million more than the usual volume of monthly submitted new claims (Mackley, 2021). Moreover, recent figures suggest that 11.7 million UK jobs were placed on furlough during the Coronavirus Job Retention Scheme between March 2020–September 2021; this provided employers grants so that staff could be retained and paid at up to 80% of their wages during COVID-19 related lockdowns (Francis-Devine, Powell, & Clark, 2021). The scale of economic disruptions has meant that many people have been exposed to forms of financial adversity during the pandemic (Legatum Institute, 2021; Witteveen, 2020).

There are concerns that the economic consequences of COVID-19 may have lasting psychological consequences for the global population (Crayne, 2020). Indeed, there is a considerable body of research detailing relationships between financial stressors and poor mental health outcomes, including distress, depression, anxiety, psychosomatic symptoms and suicide (Chang et al., 2013; Frankham et al., 2020; Hiilamo, 2020; McKee-Ryan, Song, Wanberg, & Kinicki, 2005; Milner, Page, & LaMontagne, 2013; Murphy & Athanasou, 1999; Paul & Moser, 2009; Sweet et al., 2013). Studies have also demonstrated how periods of economic recession are related to adverse mental health through job loss, financial insecurity and declining living standards (Barlow, Reeves, McKee, & Stuckler, 2015; Karanikolos et al., 2013). Moreover, there is evidence of harmful impacts on mental health and wellbeing stemming from unemployment persisting over time, a phenomenon known as psychological ‘scarring’ (Clark, Georgellis, & Sanfey, 2001).

The pathways through which financial adversity undermine health and wellbeing are thought to be driven by a number of psychosocial and behavioural mechanisms. Common consequences of financial adversity, including financial hardship (e.g. difficulties meeting daily needs, affording essentials and paying bills), have been found to engender psychosocial responses. This includes feelings of uncertainty, lack of control, stigma, shame and personal failure (Sweet, 2018a; Turunen & Hiilamo, 2014; Zurlo, Yoon, & Kim, 2014), that serve to increase stress and other adverse mental health consequences, such as depression and anxiety (Sweet et al., 2018b). In addition, financial adversity may lead to behavioural responses that directly impact health and wellbeing. For example, qualitative research has shown how periods of financial adversity can restrict the ability to source and consume food of high nutritional value (Garthwaite, Collins, & Bambra, 2015; Thompson, Smith, & Cummins, 2018), leading to risk of malnutrition (Moradi et al., 2019). People experiencing financial adversity are also more likely to be exposed to poor quality housing environments where cold or damp conditions may cause or exacerbate respiratory complications, and overcrowding can increase rates of infectious disease transmission (Gibson et al., 2011). Moreover, financial instability may mean people forego leisure and social activities ( Sweet et al., 2018b), contributing to increased loneliness, a known risk factor for poorer mental health outcomes (Leigh-Hunt et al., 2017). This body of global evidence confirms that forms of financial hardship - including debt and the inability to afford goods, facilities or services - is an important socioeconomic determinant of poor health (Allen, Balfour, Bell, & Marmot, 2014; Dalstra et al., 2005; Leung et al., 2020; Sweet et al.,2018b).

Thus far, international data has shown considerable impacts on mental health and wellbeing as a result of job loss, workload declines and worries relating to finance during the COVID-19 pandemic (Guerin, Barile, Thompson, McKnight-Eily, & Okun, 2021; Posel, Oyenubi, & Kollamparambil, 2021; Witteveen & Velthorst, 2020; Wright, Steptoe, & Fancourt, 2021). Wright et al. (2021) found that worries about finances were more strongly related to poor mental health than the actual experience of job loss, highlighting how the prospect of financial adversity is particularly burdensome (Burgard, Kalousova, & Seefeldt, 2012). Studies have also shown that financial adversity during this period increased the likelihood of being food insecure (Brown, Mills, & Albani, 2022) and being unable to consume healthy and nutritious food (Koltai, Toffolutti, McKee, & Stuckler, 2021).

International data have shown how those from lower socioeconomic and racially minoritized groups were disproportionally exposed to unemployment and job loss during this period (Hardy, Hokayem, & Roll, 2021; Witteveen, 2020), with significantly worse physical and mental health outcomes occurring as a result (Guerin et al., 2021). Indeed, there is evidence from the UK that families and individuals living on a low-income or in poverty faced additional financial burdens during this period, including new costs and expenses and the inability to enact previous strategies to ‘buffer’ the impacts of financial adversity including ‘shopping around’ for reduced items in supermarkets (Hill & Webber, 2021; Patrick et al., 2022).

Many low-income families have also had to navigate financial adversity in the context of reduced face-to-face service provision, which has interrupted access to services such as debt and financial advice charities, local welfare and social security agencies and foodbanks (Hill & Webber, 2021; Papadaki et al., 2022; Power, Page, Garthwaite, & Patrick, 2020). Foodbanks also experienced a drop in food donations and inability to purchase items due to supermarket rationing in response to public stockpiling (Butler, 2020). These issues are now being experienced against a backdrop of increased consumer prices (including energy and food) which have driven inflation levels in the UK to some of the highest recorded levels in decades (Harari, Francis-Devine, Bolton, & Keep, 2022).

Despite this emerging body of research, there is relatively little qualitative research into how specific forms of financial adversity, including loss of work, furlough and reduced hours, impacted the health and wellbeing of individuals during the pandemic. Understanding these mechanisms are crucial to the design of effective policies to mitigate harms stemming from financial strain, particular for those disproportionally at risk of unemployment and job loss during this period. Of further interest is how welfare providers are continuing to respond to these adversities in this context, including any adaptations to support measures implemented during this period. Understanding these experiences can help shape future responses to financial adversity in periods of economic instability.

To these ends, this study aimed to qualitatively explore factors affecting financial adversity and subsequent health and wellbeing of UK adults during the COVID-19 pandemic.

## 2 Methods

This study formed part of the University College London (UCL) COVID-19 Social Study (UCL, 2021), which explores the psychosocial effects of COVID-19 and associated restrictions on people living in the UK. The research was a qualitative semi-structured interview study with (1) people who self-identified as experiencing a form of financial adversity during the pandemic, and (2) service providers working in social welfare support services for people experiencing financial hardship. Interviews were conducted between May–December 2021 and focused on participant experiences of financial adversity and mental health and wellbeing impacts throughout the pandemic. Ethical approval was provided by University College London research ethics committee [Project ID 6357/002].

### 2.1 Sample and recruitment

Participants and service providers were recruited purposively via social media and the UCL COVID-19 Social Study (including its newsletter and website) (UCL, 2021). Community welfare services (e.g. community centres, foodbanks) also advertised the research via bespoke posters and fliers within service settings. Participant eligibility included (i) self-identifying as someone experiencing a form of financial adversity during the pandemic, including job loss, income reduction, difficulties paying for everyday essentials (e.g. food, utility bills, payment on credit cards or other debts) and/or experiencing any other changes in circumstances resulting in a major reduction in income since the start of the pandemic, (ii) aged over 18 and (iii) living in the UK. Inclusion criteria for service providers was (i) aged over 18 and (ii) engaged in voluntary or paid employment in a UK social welfare support service during the pandemic. Participants were purposively recruited to ensure diversity of gender, age, ethnicity, and, in the case of service providers, occupation. All participants were informed that their involvement was voluntary, and they were provided with both verbal and written information that outlined the purpose of the research. In addition, demographic details (e.g. age, gender, ethnicity) and signed informed consent were obtained from all participants and service providers.

### 2.2 Data collection

**Fig. 1:**
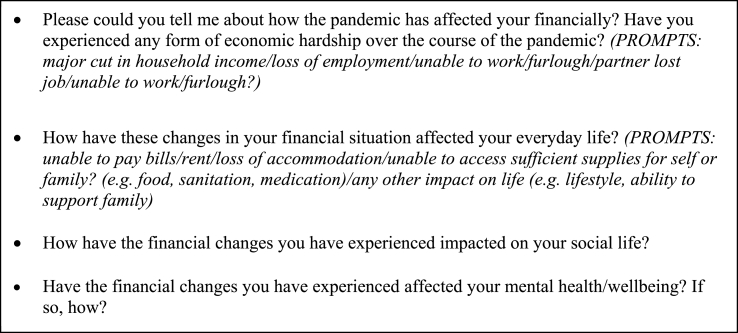
Example topic guide questions and prompts.

Interviews were conducted by TM (research fellow), AM (research fellow) and AB (senior research fellow) via telephone or video call. Interviews lasted an average of 49 ​minutes (range 27–75 ​minutes) and followed a semi-structured topic guide. This enabled data collection on the factors affecting the financial situation, mental health and wellbeing of people experiencing financial adversity during the COVID-19 pandemic (see Fig. 1 for examples of specific topic guide questions and prompts and Appendix I for full topic guides). All participants and service providers were offered compensation for their time in the form of a £10 high street voucher. The interviews were digitally recorded and subsequently transcribed verbatim by a professional transcription service with participant consent.

### 2.3 Data analysis

Following de-identification, transcripts were uploaded to NVivo version 12 software. To analyse data, a reflexive thematic analysis was used, in line with the principles of Bayes, Holley, Haycraft, and Mason (2021). This process began with two research team members (TM and HA) independently reading and coding three transcripts and then discussing any codes or emerging themes of potential significance to the research objectives. Concepts within the topic guide were used to deductively form a preliminary coding framework, and the researchers also applied an inductive approach to refine the framework as concepts within the data were identified. The remaining transcripts were analysed using the agreed framework by TM, who re-read transcripts and coded and synthesised text into categories and subsequent themes. Weekly meetings were held with all research team members, who discussed and iteratively refined new codes or themes that emerged.

## 3 Results

### 3.1 Participant characteristics

Twenty-six people took part in the study; twenty participants who experienced financial hardship during the pandemic and six service providers.

#### 3.1.1 Participants experiencing financial difficulties

Participants were aged 18–64 and predominantly white British. At the time of interview, most participants were either in part time employment or unable to work due to disability or illness. Table 1 Provides an overview of their characteristics.

**Table 1:** Characteristics of participants experiencing financial difficulties.

Demographics	mean/(range) or n
**Age**	41 (18–64)
**Gender**	
Male	10
Female	10
**Ethnicity**	
White British	11
White Other	3
Asian or Asian British Bangladeshi	1
Asian or Asian British Indian	1
Asian or Asian British Pakistani	1
Black or Black British Caribbean	1
Black or Black British African	1
Mixed Race White & Asian	1
**Employment status**	
Part time	6
Unable to work due to disability/illness	6
Unemployed and seeking work	4
Full time employment	2
Self employed	1
Student	1
**Living situation**	
Alone	6
With parent(s)	4
With partner/spouse	3
With children	2
With partner/spouse and children	2
With housemates	1
With sibling	1
With ex-partner/spouse and children	1
**Physical health conditions/disabilities**^a^	
No physical health conditions reported	10
Chronic pain disorders	3
Angina	1
Endometriosis	1
Colon cancer	1
Mobility issues	1
Graves disease	1
Osteoporosis	1
Sight impairment	1
Irritable bowel syndrome	1
Long COVID	1
**Mental health conditions/neurodevelopmental disorders**^a^	
No mental health conditions reported	8
Anxiety and depression	5
Autism	3
Bipolar disorder	2
Depression	1
Anxiety	1
Obsessive compulsive disorder	1
Seasonal affective disorder	1
Post-traumatic stress disorder	1
Dyslexia	1
Borderline personality disorder	1
Attention deficit hyperactivity disorder	1
Condition not disclosed	1

a Numbers exceed total sample number as some participants reported more than one health condition.

#### 3.1.2 Service providers

The six service providers came from a range of social welfare support services providing support to those in financial hardship, including national debt charities, citizens advice, foodbanks and a community centre providing support with employment and benefit applications. The average age of service providers was 47 years old (range 32–64); half were male and five were White British.

#### 3.1.3 Themes

**Fig. 2:**
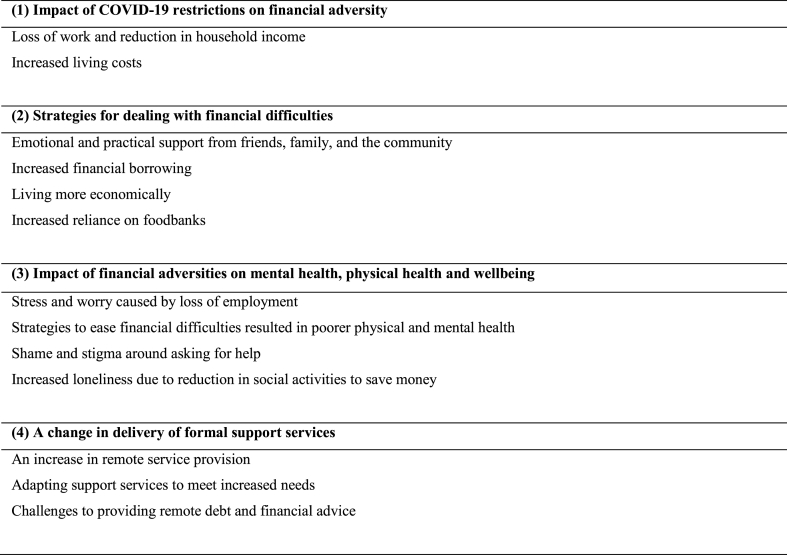
Themes and subthemes.

Four primary themes were identified: (1) Impact of COVID-19 restrictions on financial adversity, (2) Strategies for dealing with financial adversity, (3) Impact of financial adversities on mental health, physical health and wellbeing and (4) A change in delivery of formal support services. Themes are shown in Fig. 2, along with their respective subthemes.

### 3.2 Impact of COVID-19 restrictions on financial adversity

#### 3.2.1 Loss of work and reduction in household income

Although some participants reported pre-existing financial adversity due to unemployment or low incomes, others noted how the pandemic had triggered an immediate reduction in their income. This was often due to the loss of work - either for themselves or another household member (e.g. their partner) - or an acceptance of shorter hours or lower pay in new jobs following redundancy. Some also described being unable to find new employment, because they were burdened by additional responsibilities such as childcare or because of a lack of employment opportunities during this period:

*I feel it’s been harder for me to find a job … maybe, there’s more people looking for work right now, so, that’s why I think it’s harder … and I guess I look for online work as well and I’ve had no luck from there either … obviously, I think that has even more competition.* (Male1, aged 31–35)

In response to substantial decreases in household incomes, some participants applied for and claimed Universal Credit (UC) during this period. New claimants with little knowledge of the benefits system reported initial confusion regarding eligibility, and difficulties navigating the application process, an issue compounded by a lack of face-to-face contact (see subsequent theme: *3.5.3.* *Challenges to providing remote debt and financial advice*). Following assessment, some also discovered that their claims were subject to caps or deductions or affected by other household earnings (e.g. that of a partner), which decreased their entitlement. For instance, the fact that UC considers joint household income as part of its conditions, significantly reduced the amount some claimants were entitled to, especially if only one member of the household had been subject to job loss during this period. One participant described difficulties in paying for their rent as a result of this condition:

*We signed up to Universal Credit, but because we live together, and because we’re in a relationship, my income was counted as part of our joint household income. So, again, [that] took the proportions of what he would be able to do. So it also meant that we didn’t really get anything from that. We are on Universal Credit, but it was going to be £30 for the month or something, which is not enough, also, for rent.* (Female1, aged 26–30)

The inadequacy of UC payments was a common theme among participants. Indeed, those who had been subject to sudden job loss reported how a transition to UC represented a substantial decrease in income. Despite a £20 increase in the UC standard allowance to support claimants during the pandemic period, concerns about the impending removal of this temporary uplift in October 2021 were reported, particularly as many already faced difficulties living within their existing UC budgets: *“Not having money, is stressful even of itself, over the last few weeks, I've spent a lot of time worrying about UC. Especially how that's changing with this £20 increase being removed* (Female 8, aged 26–30).

#### 3.2.2 Increased living costs

Many participants reported how extended periods spent inside the home during lockdown contributed to increased energy costs and food consumption. Service providers also reported client experiences of increased costs due to spending more time at home:

*It’s made the situation worse in that people are sitting in their house 24 seven a lot of the time. And you’re using more electricity that you used. You’ll probably be eating more than you used to. You know. And trying to fill that time up. That ends up costing people money. If not through directly buying stuff, just through using electricity, gas.* (Male, Community Centre Worker, aged 51-55)

One participant suggested how even small increases in monthly payments represented a significant additional cost given their tight monthly budget:

*I’d say probably on everything at the moment, probably [an increase in costs of] about an extra £40 to £50 a month, which doesn’t sound a lot. But you times that by 15 months and that is quite a lot of money basically.* (Male3, aged 41–45)

### 3.3 Strategies for dealing with financial difficulties

As well as attempting to deal individually with financial burdens during the pandemic through cutting back on spending and non-essential items, participants also reported the use of formal and informal support networks to assist during this period including accessing foodbanks and help from friends or family.

#### 3.3.1 Emotional and practical support from friends, family, and the community

Participants described the importance of informal support provided by friends, family and the community during this period. Some reported difficulties managing financially and described borrowing from family members to meet daily needs:

*[I'm] on Universal Credit but it’s very difficult to manage. I receive it on a monthly basis, but the income doesn’t allow me to pay for all my needs. So as a result the income lasts for two weeks, and then after two weeks I just have to lend ideally from my mum.* (Male1, aged 31–35)

Although some participants expressed emotional shame at disclosing their financial precariousness to friends and family (as will be described in the theme: *3.4.* *Impact of financial adversities on mental health, physical health and wellbeing*), they still functioned as important sources of emotional and practical support. Some participants described how prior to the pandemic these networks would often provide help with food or childcare that helped alleviate some aspects of financial difficulty. Emotional support was also beneficial in buffering the emotional strain of living in hardship.

The advent of social distancing regulations, however, restricted the ability to access and engage with these networks. Some participants also grieved the closure of social spaces, including libraries and community centres, that offered practical assistance and help with job searches and benefit applications. One participant described attending the library prior to the pandemic “*About three or four times a week. It was where I could access the internet and print paperwork out”,* before its closure hampered job searching and reduced “*the only line of contact that I'd get in terms of interface*” (Male 8, aged 61–65). Participants also reported reducing expenditure on daily activities to help cope with increased living costs during the pandemic, which often meant they were unable to see family or friends during the lockdown period:

*I haven’t really been organising social stuff, as such, because it’s hard when you haven’t really got the funds. And I’m very much like I’d rather not do it if I’m going to be watching the money.* (Female4, aged 41–45)

#### 3.3.2 Increased financial borrowing

Many participants reported increased financial borrowing - either through increased spending on credit cards or overdrafts, or loans from friends or family - to help ease financial strains during the pandemic. Service providers also described accounts from service users using additional borrowing and credit to pay for daily essentials, such as mortgage repayments and bills. This method was especially prominent among study participants who reported being particularly hard hit financially by the pandemic, and service providers observed self-employed workers being particularly affected:

*I think people that were self-employed especially who almost seem to live on zero income so they’ll be behind with their mortgage and all of their bills and they will have had to take out additional borrowing and their credit rating probably wasn’t great because they are already in problem debt so they’d been getting these credit cards with really high rates of interest. Some people have been doing it to pay the mortgage, borrowing from a credit card.* (Female, Debt Advisor, aged 36–40)

Some described how the continual process of borrowing and repaying money was challenging, especially as many had experienced reductions in their income. The increased reliance on loans and credit cards therefore required constant management and planning in attempts to pay for necessities such as food:

*It’s like a juggling act, so I think my mum has a saying, robbing Peter to pay Paul, and I feel like I’m doing that half the time, because I don’t have enough money in the current account, so I’ll put it on the credit card. So, I don’t go without the food, but actually, I’m not sticking to the budgets half the time.* (Male 6, aged 31–35)

A further method of financial borrowing among participants was asking for help from friends or family. This often involved being given money, vouchers, or increased contributions toward daily living from spouses:

*So, my mum and her partner did start to issue me M&S vouchers. It could have been somewhere cheaper, but they did do Tesco vouchers as well, but they did three of these. A £50 voucher for Tesco or M&S, which did help. The same with my dad, he would say, oh, please let me know if you’re in financial difficulty.* (Male7, aged 26–30)

#### 3.3.3 Living more economically

The increased costs associated with more time being spent at home meant participants often enacted a range of strategies to help them cope with the additional financial burden during this period. The most common of these were a transition to cheaper and non-branded/supermarket own brand products and a reduction in food purchasing, consumption or skipping meals entirely as described by a single parent: “*There were some months having to try and manage, and then being like okay, well today I might only be able to have one meal a day*” (Female 5, aged 36–40).

Several participants also reported reducing frequency of laundry or washing, often choosing to wear unclean clothes or wash items by more burdensome methods to save money *“During the pandemic, I would always do handwashing, I couldn't afford washing”* (Male5, aged 51–55). Some reported denying themselves non-essential items in attempts to save money: “*We couldn't afford the little treats that you buy every now and again. And things like that were having to stop”* (Male2, aged 51–55). One participant reported how her efforts to remain within financial budgets often came at the expense of other luxuries which, at times, put additional strain on participant relationships:

*There would be situations where my partner would say oh, shall we get a takeaway this weekend? I would want one, and then I would look up and be like no, we can’t, because we can’t afford it. We’ll have to wait until I get paid, and then you can have it. The money hasn’t gone through yet. So it ended up being a really strange power dynamic where he, as a grown man, would have to ask me for takeaway and for food and stuff. And I’d have to say no, which we both hated and we’re both pretty aware it’s not what we want.* (Female1, aged 26–30)

#### 3.3.4 Increased reliance on foodbanks

Foodbanks reported a major increase in demand during the pandemic, often linked to a range of pandemic specific problems, including immediate and sudden job losses, limited availability of low-cost food and other goods due to people stockpiling items, and inadequate financial and social protection:

*It was people who had worked in hospitality cleaning, etcetera, and weren’t furloughed, either couldn’t claim benefits or didn’t know anything about the benefit system.* (Female, Foodbank Volunteer, aged 51–55)

Similar to service provider accounts, some participants reported an increased reliance on foodbanks during this period compared to previously. Reasons for doing so were varied, with some using them in direct response to COVID initiated adversity, including loss of employment, income loss, rising living costs and the closure of supermarkets “*it was increasing credit, not being able to go to the supermarket and get stuff. And, yes, I found these foodbanks, who do food parcels, they were really, really supportive through COVID”* (Male3, aged 41–45), whilst others viewed them as a resource though which to subsidise increased costs such as heating bills during this period:

*So in terms of foodbanks, I’ve had to visit them because they’re the priority where there’s not enough income to subsidise that, so you have to find out initiatives and other programmes where you cansavecosts, you can benefit. So the foodbank has been helpful in enabling me to get access to resources.* (Male1, aged 31–35)

### 3.4 Impact of financial adversities on mental health, physical health, and wellbeing

Participants described both the mental and physical health toll of the financial adversities they were experiencing, as well as the negative impact of some of the strategies they were using to cope on their health and wellbeing.

#### 3.4.1 Stress and worry caused by loss of employment

Participant narratives highlighted how the immediate loss of employment was a stressful and worrying experience, which impacted on their mental and physical health. Whilst some described how employment inactivity led to “*just like pure boredom”* (Male5, aged 51–55), and a lack of routine and purpose: “*not having work, on top of that there are no routines, and then I feel like what is my sense of purpose?”* (Male1, aged 31–35), the financial consequences appeared to have the greatest bearing on wellbeing. Many participants had worries about how reductions in income would lead to difficulties to support oneself: “*Well, if I run out of money, I won't be able to get food”* (Male3, aged 41–45) and the ability to stick within pre-existing household budgets: “*We were both really stressed and worried about this. We'd go to bed every night, really worrying about our budget and looking at the budget every single day of whether we could afford things”* (Female1, aged 26–30). The emotional burden of a reduction in household income often manifested itself through feelings of stress and anxiety as described by a participant who was furloughed during the pandemic:

*I still feel stressed, to be honest with you. I’m stressed because of the fact of the money, and I know that we’re not getting on top of that. It’s still going to be a long time before we do get on top of it. So, that stresses me out.* (Male2, aged 51–55)

Several participants described how they or their partner had lost work, with the subsequent reduction in income triggering periods of excessive rumination and worry. For some, this often led to physical symptoms such as sleep problems and a sense of detachment that impacted on their daily life:

*And I used to lie in bed, and then have a thought about some sort of figure in our bank account or our budget or whatever, and then have to look it up on my phone in the middle of the night. Otherwise, I definitely couldn’t get it out of my head. So I think, definitely, in terms of physical manifestations, yes, lack of sleep. But yes, I used to sit feeling, especially at my lowest point, on edge the whole time, not feeling very present with chatting to friends and stuff as well. If I had a Zoom call with friends, not feeling like I can focus on them.* (Female1, aged 26–30)

Others reported how the severity of COVID-19 restrictions and a perceived lack of control over the situation contributed to feelings of uncertainty. This was often linked to worries about the long-term consequences of reductions in household income and how this might affect plans for the future:

*So, there’s a semi-permanent worry, at the minute, about what we do in the longer term … I don’t know what the winter looks like, I don’t know what next year looks like, I don’t know if we’ll be able to go back to our lifestyle, I just don’t know what will happen. And I think that uncertainty is the biggest problem at the moment.* (Female 3, aged 41–45)

#### 3.4.2 Strategies to ease financial difficulties resulted in poorer physical and mental health

Whilst reducing the consumption and purchase of food eased financial burdens, many participants described how such behaviours often had direct implications for physical health. A greater reliance on cheaper, tinned food, for example, made maintaining a healthy diet challenging:

*Yes, we don’t have all the right nutrients, vitamins, all the rest of it, we’re more tired. I think we’re more anxious, we don’t really go anywhere, do anything. We’re housebound a lot, that sort of thing. Imagine living off cans constantly. That’s your only choice, we can’t really afford to buy all the stuff.* (Female 6, aged 26–30)

Some, particularly those with pre-existing health conditions, reported deteriorations in their health or an exacerbation of conditions in response to consuming poor quality or a limited range of food:

*And I felt it. To be honest with you, I have a stomach ulcer, and so, I must be careful what I eat. And I was buying exceedingly inferior food I know I shouldn’t be eating … [it] meant that eventually I did lose a considerable amount of weight ….it was down to bananas and crisps, to be frank.* (Male4, aged 51–55)

Despite being grateful for the food they were provided with by foodbanks, some participants noted a lack of nutritious and culturally appropriate food, meaning they instead had to rely on suboptimal alternatives:

*We’re grateful, I’m not going to lie, but at the same time we’re not really grateful because, no offence, it’s kind of white people’s things and it’s not really catering to our needs. Let me explain something. So we’re from [Asian] background and they just give us cans all the time … cans of stuff, half of it we can’t eat, it’s different tastes to our needs.* (Female6, aged 26–30)

Participants also detailed how increased financial borrowing and the accrued debt they generated, was often a stressful and burdensome experience, with descriptions of *“building debt”* weighing heavily on them. Worries relating to increased debt were often exacerbated by feelings of being ‘trapped’ in perpetual borrowing cycles that characterised periods of indebtedness. Some participants described how increases in debt and spending of savings were experienced as overwhelming and contributed to emotional and psychological pain. This also led to some participant's engaging in behaviours such as taking more drugs and spending more money to cope with these negative feelings:

*So as of a couple of months ago there wasn’t any more savings. And the text from the bank that says, you have gone into your unplanned overdraft is what’s been happening. And I’ve got really depressed and sad and emotional and took more drugs because … yes. You kind of go, oh for fuck’s sake, what’s the point?* (Female7, aged 36–40)

The challenge of repaying loans often contributed to behaviours that were likely to be detrimental to physical health and wellbeing. One participant making use of a high-interest, payday loan service described how the structuring of the loan left him short of money once he had paid off the borrowed credit. This resulted in him being unable to afford sufficient amounts of food during these periods, which led to him skipping meals:

*During lockdown there’d been three or four occasions where I’ve got really, really short of money and had to borrow. And if I borrow from these people, the minute I get any money again, it could be my UC (Universal Credit). They see a credit go in and rather than just taking like a £100, if I owed £400, as soon as my UC went in, they would take the full amount back … I would then have to borrow that money back again …. But there have been times where it’s got to a point where the money’s gone. And it’s about three or four days until I get my next lot of money through. So, I just find myself not eating properly, just snacking on stuff.* (Male3, aged 41–45)

Deferring payment of bills to save money during this period helped alleviate short term concerns and allowed money to be redirected elsewhere, however participants expected future consequences. Heightened anxiety about potential consequences were widespread in participant narratives. Fears of potential debt collection left people feeling *“on edge”* (Female1, aged 26–30):

*Because what was happening was the seconds were turning into minutes, the minutes into hours and hours into weeks. And so, every time the phone rang, you expected the worst. In other words, is it going to be a bailiff … Was it going to be a debt collector? Was it going to be this? Was it going to be that? Am I going to sleep in the street tonight?* (Male4, aged 51–55)

#### 3.4.3 Shame and stigma around asking for help

Whilst receiving financial support from family or friends helped alleviate feelings of financial pressure, having to ask for help often prompted feelings of shame and guilt. For some, asking for financial help and therefore disclosing being in a position of financial hardship prompted these feelings. Such sentiments were widespread in participant narratives, with one participant describing how “*I feel really, really bad asking people to help me out*” (Male3, aged 41–45). Others, particularly those who had been financially comfortable prior to the pandemic, found the experience of asking and receiving financial support particularly uncomfortable given the perceived relative status of their financial position in comparison to others:

*I feel really awkward saying that I am in that position, particularly for like, I have a smart phone, and I speak to people, when I do the mutual aid stuff, who are really in financial difficulty, and we’re experiencing different types of financial insecurity, and I just don’t think it’s ethical for me to say that I’m in a relative position to those types of people.* (Male7, aged 26–30)

Some participants had worries about the social stigma of foodbank use, which often made it hard for them to accept help. This was most noticeable among those who were using services for the first time, who described a reluctance and hesitancy to enter foodbank settings due to a sense of shame and failure:

*I went to the one which was 15, 20 minutes away and I stood in the queue … And you felt belittled. You felt this is not what I expected … I felt sad that I had got to that position.(Male4,aged51–55)*

#### 3.4.4 Increased loneliness due to reduction in social activities to save money

Financial adversity coupled with social distancing restrictions meant a loss of social networks and a reduction in practical and emotional support. Having to limit social activities impacted mental health and wellbeing through feelings of loneliness and isolation due to not being able to connect with friends:

*The pandemic has created some mental health difficulties where I’ve had to think twice, it’s put me on edge to some extent. Where I was working before I could pay out my rent, I was living comfortably.But now I’ve had to claim Universal Credit welfare assistance where it’s placing an impact on my mental health. Because I’m not able to meet friends, or go out for that meal, or go places outside, go on a holiday, it’s just not possible anymore.* (Male1, aged 31–35)

Isolation was felt particularly strongly among those with pre-existing mental health conditions, which were often exacerbated by both financial adversity and the conditions of the pandemic:

*I felt really, really isolated. And I’ve noticed a drop in my mood. And back in March, my doctor had to increase my antidepressants, because I was really, really struggling with lockdown ….It was the financial strain, not seeing people, not being able to meet up with people as well. And I just felt really, really isolated.* (Male3, aged 41–45)

### 3.5 A change in delivery of formal support services

Participants described accessing a range of support and services during the pandemic to assist with the financial and mental health implications of lockdown including debt advice charities, mental health support and community hubs. Service provision changed from predominantly in-person to remote delivery and these changes were sometimes experienced as challenging by both participants and service providers.

#### 3.5.1 An increase in remote service provision

The pandemic had a major impact on the way that many financial support services were delivered. While some forms of telephone advice were available prior to the pandemic, many services, particularly those providing financial advice, responded to social distancing measures by transitioning to remote-only operations, including telephone or video calls for assessments and support. In a context of limited face-to-face services, providers felt that such methods offered unique forms of support that were particularly beneficial during this period. Indeed, some services described expanding their operations, including providing “*24-hour* *service, rather than nine to five*” (Male, Debt Advisor, aged 31–35)*,* telephone services and dedicated COVID-19 hotlines that increased service user connectivity. One foodbank worker reported how remote service provision also facilitated greater possibilities for multi-agency working. This enabled service users to be referred to services that could respond to specific challenges, for example when service providers had concerns about a service user's mental health:

*So these two Covid helplines or referral helplines is what we gave to everyone, and they were really helpful in keeping people connected. The local one … it wasn’t just with foodbank referrals, but it was also arranging a volunteer who would go and get your medication, or a personal shopping service, or a befriender, and just connecting people withother support like mental health support andso on, so that’s been a really good thing that has come out of the crisis that we’ve now got this telephone line which is quite well used actually. That was really important, and it was really important as well in getting key organisations in* < *area> talking to each other and trying to problem solve together.(Female, Foodbank Volunteer,aged51–55)*

#### 3.5.2 Adapting support services to meet increased needs

Service providers described various pandemic-related problems that altered the type of support they offered during this period. Some observed an increase in isolation and loneliness among service users, which necessitated nuanced and more considered responses. For instance, one service provider described how “*some people were really wanting to talk as well, you could tell from these long conversations, some people really needed some contact*” (Female, Foodbank Volunteer, aged 51–55). In response, some service providers described offering more informal support over the phone during this period:

*I did feel a bit more during lockdown obviously especially elderly people are quite isolated and lonely so you would feel people did want to keep you on the phone and have a chat, that’s fine. Sometimes I wanted to keep them on the phone for a chat … so you’d have more of a chat with people, you pick up on that, people are just needing a bit of a chat.* (Female, Debt Advisor, aged 36–40)

Service providers noted how their work had become more reactive and were now offering forms of support and assistance that were responsive to the issues reported by service users. Whilst this included initiatives serving the immediate needs of service users such as provision of household items, clothes and hygiene products “*the two run together, so they get foodbank and things like shower gel, shampoo, stuff for the washing machine, washing up liquid, loo roll*” (Female, Foodbank Volunteer, aged 61–65), additional measures attending to the drivers of financial hardship (e.g. job loss, financial instability) were also considered. One foodbank worker described how these issues were now being catered to through increased financial support services in foodbank settings, which were delivered in a context of reduced availability of more conventional financial support services during this period:

*I think the big thing at the beginning was sort of in April, May last year was accessing Citizens Advice service, that was really overrun … We’ve now got advice workers in our centres, and when we say to people, “Go and deal with your debt, there’s an advice worker just sitting there, go and have a conversation,” people say, “Oh, I wish you’d told me that months ago.” I think there has been an almost giving up on seeking help and advice, and people have got into a deeper rut.* (Female, Foodbank Volunteer aged 51-55)

Foodbank workers also described setting up mobile services either outside of buildings that were closed to the public or by offering food delivery services to people's homes to avoid the spread of COVID-19:

*In March last year, we had to close our buildings because our collection centres tended to be in church buildings, they suddenly closed, so we did some mobile foodbanks on the street outside, and then from April until September last year, we were just delivering food.(Female,Project Manager,* aged *51–55)*

Others employed alternative methods to foster levels of trust and reduce stigma among new service users. One foodbank worker described how she would allow service users to contact her directly and then deliver foodbank packages to them herself:

*I found the best way, then, to contact them, was to let them have my mobile number and they could text me. They found it easier to send me a message saying, have you got any food this week, or, I’ve run out of nappies or whatever, than me ringing them and saying, what would you like? They find it very hard to articulate that, and that works much better.* (Female, Foodbank Volunteer, aged 61–65)

Another foodbank worker described how pre-existing mistrust of authorities meant some communities were hesitant to attend foodbank settings where they were required to leave their details. As a result, anonymous collections were permitted to increase accessibility:

*The Latin American community, at the beginning we found out they didn’t want to give their details. There was a kind of fear of authorities and what would be done with the information, so some people didn’t want to give their names and addresses, so we set up an informal foodbank where people could just give their name as unknown or address as unknown or something, and then our worker has over a period of months built up the confidence and the trust of those people so that they will give their details and they understand that nothing is going to happen to their details, we’re not going to pass it on to any authorities, and it’s basically safe. There was a whole relationship building, trust building exercise that had to go on with some people in that community as well.* (Female, Foodbank Volunteer, aged 51–55)

#### 3.5.3 Challenges to providing remote debt and financial advice

Despite the reported benefits of remote service provision, several challenges were noted for both service users and providers. Firstly, a lack of face-to-face services made it difficult for service providers to respond to the often complex and multi-dimensional nature of service user issues. This was a particular problem for those with specific needs, including learning difficulties, language barriers or hearing impairments:

*I’ve had service users … who do have letters they don’t understand, letters they struggle to read whether because they’re dyslexic, just don’t read well or don’t read English well. If you’re trying to do an interpreting service, you know, if you’ve got somebody who speaks no English and are doing it through an interpreter, that’s fine, until you get to the point where you say “you got that letter, what does it say?” And then you’re a bit stuck ….I mean you can if service users have e-mail access you can ask them to take a photo and send it in. They often don’t do that. Or they send the first page and you need more than that, so you have to create the back and forth that takes quite a bit of time … so that means the advice that I’m giving is based on a lot less certain information than it should be.* (Male, Debt Advisor, aged 31–35)

Similar issues were often felt most among those participants who had found themselves in hardship for the first time and with limited knowledge of what support was available. Some participants described application processes for UC as convoluted and overly bureaucratic: “*Honestly, there's so much red tape around it but it's so frustrating. So, I've spent the last four weeks stressing about all this and having so many conversations”* (Female4, aged 41–45), an occurrence which was made even more difficult given the lack of face-to-face support.

Difficulties establishing emotional connections were also described by both participants and service providers, which made such methods limited in comparison to previous physical interactions and support:

*I think there’s a type of person who wants to look them in the eye and get that rapport. I can think of a couple of service users who I did have an initial appointment with in-person. After that we did everything by phone, but they really wanted that first appointment to be in person so they could assess what I was like, whether they trusted me, which is fair enough.* (Male, Debt Advisor, aged 31-35)

The costs of electronic equipment and phone/internet bills presented a further barrier to receiving help and support. Participants previously reliant on community centres or hubs for internet use reported access issues, especially if they were unable to afford, or had difficulties accessing, wi-fi at home. This often acted as a barrier to accessing help and support:

*People who don’t have access to, that is another thing as well. I lost my Wi-Fi, couldn’t afford to pay the Wi-Fi, halfway through lockdown … there’s [also] a huge balcony above me which messes with my signal. So I have no signal now.* (Female7, aged 36–40)

## 4 Discussion

This study provides new qualitative insights into the impact of different forms of financial adversity on the health and wellbeing of people during the COVID-19 pandemic in the UK. This includes how the pandemic has exacerbated pre-existing financial stressors, and why they are detrimental to physical and mental health. Our study compliments and supports existing quantitative literature documenting how worries about finance and financial adversities directly caused by the pandemic – including income reduction and loss of paid work – are closely related to poor mental health outcomes, including anxiety and stress (Guerin et al., 2021; Kim, 2021; Posel et al., 2021; Witteveen & Velthorst, 2020; Wright et al., 2021). This is also in congruence with evidence linking previous societal upheavals such as recession to substantial psychological distress (Barlow et al., 2015; Karanikolos et al., 2013) including heightened insecurity and stress relating to changes in financial situation (Hiswåls, Marttila, Mälstam, & Macassa, 2017) and feelings of identity loss and powerlessness stemming from unemployment (Macassa, Rodrigues, Barros, & Marttila, 2021).

Our work also adds to a growing body of qualitative evidence from the UK documenting the experiences of those living on a low-income during the pandemic (Hill & Webber, 2021; Patrick et al., 2022; Pybus et al., 2021). However, our work extends these findings by exploring the pathways through which experiences of financial adversity undermine health and wellbeing, and why they are particularly burdensome during this period. For instance, many participants in our study experienced job losses in the absence of traditional supports and strategies used to buffer financial adversity and related mental health difficulties. Social opportunities and access to social networks were constrained by social distancing restrictions, which limited any possibilities of informal social support that have proved beneficial to mental health among people experiencing job loss during previous financial recessions (Macassa et al., 2021). This has also meant that many people facing financial adversity have been unable to access financial support from these networks during the pandemic (Patrick et al., 2022).

Extended periods spent inside the home during lockdown contributed to increased expenditure on energy and food consumption, which prompted additional ‘squeezes’ on finances. Whilst there is evidence that some households were able to save money during the pandemic (Handscombe & Judge, 2020), the experience was differential, with those on lower-incomes or experiencing pre-pandemic financial hardship more likely to face additional financial adversities or new costs (e.g. childcare) (Brewer & Patrick, 2021).

The study also adds to the growing literature on how people are navigating financial adversity through increasingly harmful means (Sweet et al., 2018c), which has utility for developing future psychosocial and economic support for this population. Increased financial burdens meant participants often enacted a range of behaviours and strategies in attempts to live more economically during this period that have been found to be predictive of poor health. For instance, some participants described resorting to the use of short-term, payday loans, which are particularly burdensome forms of debt with specific risks for anxiety and poor health because of their ability to trap users in perpetual borrowing cycles of high-interest debt (Sweet et al.,2018b). The finding that people are engaging in such methods during this period, perhaps in the absence of more traditional financial management strategies (Hill & Webber, 2021), is concerning given how increased financial borrowing and subsequent accrued debt is considered a strong predictor of poor mental and physical health, including perceived stress and depression (Hojman, Miranda, & Ruiz-Tagle, 2016; Zurlo et al., 2014). The projected increases in living costs in the UK- including increased food costs and rising energy bill prices - are therefore an unwelcome development and come at a time when people may be particularly susceptible to financial vulnerability and harmful coping strategies, including the use of payday loans (Mogaji, 2020).

Our findings also provide some evidence of the pathways through which financial adversities impact physical health, specifically, by altering the ability to care for oneself and maintain a healthy lifestyle. For instance, some participants described how food budgets were often deprioritised due to its greater flexibility within tight budgets compared with other expenditures (e.g. housing and fuel) (Anderson, White, & Finney, 2012; Dowler & Lambie-Mumford, 2015). Often, this involved sourcing cheaper products of inferior quality or foregoing food completely. This supports quantitative findings documenting how COVID-induced financial adversity has increased the probability of experiencing food insecurity during this period (Brown et al., 2022), including not being able to consume healthy and nutritious food (Koltai et al., 2021). Such methods are problematic given the residual effect of food insecurity on health, with families experiencing food poverty more likely to experience poor health, including malnutrition (Moradi et al., 2019) and chronic health conditions (e.g. diabetes, hypertension) (Pérez-Escamilla, Villalpando, Shamah-Levy, & Méndez-Gómez Humarán, 2014; Seligman, Bindman, Vittinghoff, Kanaya, & Kushel, 2007). Foodbanks were also utilised, although similar problems related to maintaining a balanced diet were reported. Unfortunately, this is a common problem given how foodbanks are often limited by the donations and contexts in which they operate in, including during periods of increased demand (Garthwaite et al., 2015; Thompson et al., 2018).

Finally, some participants reported challenges to accessing financial support and practical advice. Access to financial support measures, including UC, has long been considered a complex, confusing and frustrating experience for claimants (Cheetham, Moffatt, Addison, & Wiseman, 2019) and the experiences of those reported in this article are not dissimilar from other qualitative studies during this period regarding conditionality and a lack of face-to-face provision (Power et al., 2020). We add further insight here, detailing how access to financial support was particularly challenging for first-time claimants, who were frequently unaware of what support they were entitled to, and often described a reluctance and hesitancy to seek financial support due to a sense of shame. Services providing debt advice also reported difficulties with providing advice online and noted issues with the quality of remote service provision and difficulties with responding to clients with often complex needs. These findings are troublesome given the recent recommissioning of debt services in the UK, which, although likely to increase accessibility, will substantially reduce the amount of free face-to-face advice available (Bond, 2021).

This study does have several limitations. First, the sample may be biased in favour of participants motivated or willing/able to participate. It is possible that the findings presented in this paper are therefore missing the views of individuals most acutely impacted by financial adversity including those who may not have the resources to contact the study team or conduct remote telephone/video interviews. Remote interviews also have some limitations over in-person interactions, for example we did experience some connectivity issues and it can be more difficult to pick up non-verbal cues. Second, our study sample is predominantly white British, despite efforts to recruit a broad and inclusive sample. This omission means our sample only includes limited insights from Black, Asian and minority ethnic populations who are disproportionally affected by socioeconomic and health inequalities, and whose experiences of financial adversity during the pandemic may vary in ways that are not fully captured in this research. Third, the study team experienced difficulties recruiting service providers to take part, which may be due, in part, to their increased workload during this period. It is possible that a larger sample of this population would have resulted in additional insights and experiences. Fourth, we did not collect standardised objective measures on the types of financial adversity people were experiencing and whether these experiences pre-dated the pandemic. Finally, interviews were conducted between May–December 2021, a period where the most restrictive COVID-19 restrictions in the UK were not in place. This included the removal of social distancing and social contact limits and the reopening of businesses. Therefore, the timing of interviews requires consideration when interpreting the findings, although participants did recount retrospective experiences during periods when more restrictive social distancing measures were in place (e.g. stay at home orders).

Nevertheless, our study has some important implications for policy and organisational practices. First, we identified increased forms of financial adversity and subsequent reliance on strategies (e.g. increased credit and payday loans, food rationing and foodbank use) that have previously been identified as risk factors for and exacerbators of psychological distress (Sweet et al.,2018c; Thompson et al., 2018). This highlights the importance of implementing measures attending to the intersections of mental health, financial insecurity and unemployment, including the provision of accessible psychological and financial services (including financial counselling and employment support) that can identify, signpost and address the underlying drivers of financial insecurity (Bourova, Ramsay, & Ali, 2019) and have the potential to protect the mental health of those experiencing financial adversity (McGrath et al., 2021). This is particularly important for populations most vulnerable to financial adversity, including communities experiencing chronic financial hardship or who may not usually seek help with financial strain (Cheetham et al., 2019). Second, the complexities of navigating the benefits system and accessibility issues highlighted here are likely to represent significant barriers to support for the most vulnerable, who may face additional digital exclusion issues and support needs requiring targeted and specialised provision. Looking ahead, ensuring clear messaging and access to support is therefore essential to limit pre-existing socioeconomic disparities. This includes - in a context of proposed changes to debt advice in the UK, including cuts to face-to-face support - continued in-person debt advice for those with complex or multi-dimensional needs, who may require additional support (Bond, 2021). If elements of remote provision are to be continued, however, it is essential that service providers are also equipped and comfortable with using these methods and suitably supported given the risks to well-being associated with operating in this way, including increased isolation and disconnection (Edmiston et al., 2021).

Finally, our finding that many participants experienced difficulties (financially and psychologically) living on UC during this period adds to previous research highlighting the challenges of living on benefit payments, including the detrimental impacts this has on mental health and wellbeing (Cheetham et al., 2019). That many participants experienced issues with meeting daily needs prior to the removal of the temporary £20 uplift in UC payments in October 2021 is particularly concerning and highlights the need for wider consideration of the adequacy of existing rates of social security (Bannister, 2021). Indeed, many of the strategies and behavioural changes in response to financial adversity – including increased use of credit and financial borrowing and foodbank use – are often enacted to fill gaps in social provision in the context of welfare reform, austerity measures and inadequate social security (Lambie-Mumford, 2018; Riches, 2002). We therefore emphasize the need for additional policy measures (e.g. increased financial and social protection and a reduction in poor-quality and precarious employment (Marmot, Allen, Goldblatt, Herd, & Morrison, 2020)) that attend to the inequities and social-structural factors that drive financial hardship and reduce the need to engage in often substandard or even harmful coping measures.

## 5 Conclusion

This study highlights financial adversities during the COVID-19 pandemic in the UK and the subsequent impacts on mental health and wellbeing. Participants reported being exposed to multiple and intersecting financial burdens during this period - including the permanent or temporary loss of employment and elevated living costs and expenditure - that functioned as pathways through which financial adversity impacted on health and wellbeing. These factors also induced behaviour changes that are predictive of poor health, including the increased use of credit and financial borrowing, and foregoing food. Therefore, this study contributes to understanding financial insecurity as a social determinant of health, specifically within the context of the COVID-19 pandemic, with financial stressors acting as risk factors for poor health. These findings underline the importance of measures attending to the immediate needs of individuals, including accessible, co-located financial and psychological services, as well as broader measures that seek to reduce social and economic inequalities. These are especially important given the potentially lasting financial impacts of the COVID-19 pandemic on people's health and wellbeing.

## Funding

The COVID-19 Social Study was funded by the 10.13039/501100000279Nuffield Foundation (WEL/FR-000022583), but the views expressed here are those of the authors. The study was also supported by the MARCH Mental Health Network funded by the Cross Disciplinary Mental Health Network Plus initiative supported by 10.13039/100014013UK Research and Innovation (ES /S002588/1) and by the 10.13039/100010269Wellcome Trust (221400/Z/20/Z). DF was funded by the 10.13039/100010269Wellcome Trust (205407/Z/16/Z).

## Authorship

AB and DF contributed to the conception and design of the study. TM was responsible for data collection and wrote the first draft of the manuscript. TM and HA performed the formal data analysis. AB, DF, and HA assisted with review and editing. All authors contributed to manuscript revision, read, and approved the submitted version.

## Data availability statement

The data presented in this article are not readily available because they contain information that could compromise the privacy of research participants. Requests to access the data should be directed to d.fancourt@ucl.ac.uk.

## Ethics approval statement

Ethical approval to conduct the study was provided by University College London research ethics committee [Project ID 6357/002].

## Patient consent statement

All study participants provided written informed consent to take part in the study.

## Referee suggestions

Dr Mandy Cheetham, Northumbria University: m.cheetham@tees.ac.uk Research specialities: qualitative research methods, place-based and community-led approaches to addressing inequalities in health, and the health and social impacts of welfare reform, including Universal Credit.

Dr Daniel Edmiston, University of Leeds: D.Edmiston@leeds.ac.uk Research specialities: poverty and inequality; welfare; citizenship; social security and activation policy; public service reform; social innovation; social impact investment.

Dr Shamini Gnani, Imperial College London: s.gnani@imperial.ac.uk Research specialities: primary care, public mental health, quality improvement and health inequalities.

## Declaration of competing interest

The authors declare that they have no known competing financial interests or personal relationships that could have appeared to influence the work reported in this paper.
