# Supplementary material for: Financial adversity and subsequent health and wellbeing during the COVID-19 pandemic in the UK: A qualitative interview study
Source: SSM Qual Res Health. 2023 Jun;3:100224. doi: 10.1016/j.ssmqr.2023.100224 (PMC9883074; doi:10.1016/j.ssmqr.2023.100224)
Supplement: Multimedia component 1 [file mmc1.docx]

**Interview topic guides**

**Participants experiencing financial difficulties**

**Ask to describe ‘normal life’**

- Employed? Type of job, hours etc,
- Living situation
  - Who you normally live with, does this change, separated/ extended family?
  - Has this accommodation changed recently?
- Education/study?
- Full time parent or carer?
- Use of any community services?
- If they have any health conditions they mentioned (what condition, when diagnosed,

if on or whether they have had complete treatment)

- Whether you would usually have done any type(s) of regular exercise (whatever they

perceive as exercise including walking/gardening)

- Are you/have you been registered with any support services? (third sector etc).

**At the moment, are you self-isolating (how long for, reasons for this) a key worker,**

**working but not a key worker, social distancing/ ‘staying at home’**

- Please describe what this is for you and your family/ household?
  - i.e. are you self-isolating with neighbours helping to get groceries, or going out for these?
  - Or self-isolating with no outside exercise? Etc – if self-isolating without outside exercise how are you feeling about this? Do you have a garden or outdoor space?

**What do you understand by the ‘social distancing’ advice that is being given –what does it mean to you?** Have you been…

- Avoiding crowds
- Keeping personal distance from others
- Isolating
- Avoiding close contact greetings
- Socialising/going out only with those in your household
- <if exercising outside> are you finding places to go where you can keep your
- distance from others?

**Have you been able to stick to the social distancing advice that has been given to**

**your group? Please tell us about why/ why not? [COM-B prompts can be used here, to include:]**

- Have your experiences (e.g. change in financial circumstances) had any impact on being able to follow social distancing guidelines?
- Any existing physical or mental health problems
- Group membership/ applicability
- Beliefs about consequences/ health beliefs
- Consequences for others/ self
- Needing to work/ living arrangements, whether others are self-distancing in the same house/area
- Work/ Caring responsibilities, providing emotional support
- Peer pressure to socialise
- Government rules/punishments <prompt to ask how they feel about the Government recommendations that are relevant to exercise for them>
- Feelings about losing normal life
- Change of routine/ habits
- Any impact on your ability to access support?

**Please could you tell me about how the pandemic has affected your financially? Have you experienced any form of economic hardship over the course of the pandemic?**

- Major cut in household income?
- Loss of employment?
- Unable to work/furlough?
- Partner lost job/unable to work/furlough?
- How have these changes in your financial situation affected your everyday life?
- Unable to pay bills?
- Unable to pay rent?
- Loss of accommodation?
- Unable to access sufficient supplies for self or family? (e.g. food, sanitation,
- medication)
- Any other impact on life? (e.g. lifestyle, ability to support family)

**How has Covid-19 had an impact on the [health condition] (if applicable)?**

- What has been the impact on any normal appointments? (cancelled, delayed, unable
- to speak to appropriate healthcare professional, changed to different method/location
- of appointment e.g. online/telephone)
- What has been the impact on any treatment? (cancelled, delayed, changed from
- usual treatment plan, unable to get medication/prescriptions)
- Have you experienced an impact on any symptoms/side effects?
- How have you felt about [any mentioned changes/impact above]?

**How would you describe your social life before the Covid-19 pandemic?**

- How would you describe your social network – for example size, types of people,

types of relationships, do they live with you, nearby or further away, how often do you

see each other, how well do you know each other? How do you interact, face to face,

online or social media?

- Social activities?
- Could you describe any community services/participation or volunteering

participation?

- Could you describe the social support you have? (such as emotional support, advice

and information, someone to help you with money or milk/bread/essentials,

community services)

- Can you tell us about any ways your social networks/ friendship groups influence

you, such as peer pressure, or encouraging you to get involved in things? Do you

compare your life to theirs?

- Social engagement (social roles, bonding, attachment)

**How would you describe your social life now that social distancing measures have**

**been brought in because of Covid-19? Please tell us about this**

- How would you describe your social network – for example size, types of people,

types of relationships, do they live with you, nearby or further away, how often do you

see each other, how well do you know each other? How do you interact, face to face,

online or social media?

- Social activities?
- Could you describe any community services/participation or volunteering

participation?

- Could you describe the social support you have? (such as emotional support, advice

and information, someone to help you with money or milk/bread/essentials, getting

medication/access to healthcare, community services)

- Can you tell us about any ways your social networks/ friendship groups influence

you, such as peer pressure, or encouraging you to get involved in things? Do you

compare your life to theirs?

- Social engagement (social roles, bonding, attachment)
- How have the financial changes you have experienced impacted on your social life?

**How do you feel about the changes that have been brought about by Covid-19?**

**Have they had any impact on your mental health or wellbeing? Please tell us about**

**these**

- What are the things most bothering you at the moment?
- Have you experienced any impact on positive emotions? (prompts: how deeply you
- can engage with what you are doing, sense of meaning/ purpose, relationships with
- others, how well you are managing and feelings of control over your situation?)
- Has there been any impact on your sense of identity?
- Have you experienced any negative psychological feelings? (prompts: such as
- shame, guilt, lack of pleasure, anxiety, worry)
- Please tell us about any physical symptoms due to being stressed or anxious?
- (prompts: fatigue, sleep problems, pain, illness symptoms, palpitations)
- Have the financial changes you have experienced affected your mental health and
- wellbeing? If so, how?

**Have you been doing/ planning anything to help with this?**

- Connecting with family or friends/ work colleagues online?
- Online groups?
- Hobbies/ Reading
- Exercise at home <ask about what they have been doing and if there are specific
- resources they have found useful to exercise>
- Volunteering
- Other engagement

**Why are you doing/ not doing these things?**

- Helpful/ not helpful – please tell us why
- Enjoyable
- Good for mental health/ wellbeing
- Can’t get online, not connected, not comfortable, affordability, confidence in using/
- skills
- Skills in using the internet/ communication software
- Living arrangements/ Work/ caring demands
- Peer support/ pressure
- Difficulties/ restriction in physical environment

PROSPECTION

**Has the pandemic/changes in financial circumstances meant that you have any**

**worries for the future?**

**How are these different from the worries you had before?**

**Will this change the way you live your life in future?**

- The way you connect with others
- How you look after yourself
- How you support others
- How you work?
- How you exercise?

**Has this changed any of your priorities for the future?**

**Welfare Providers (Financial support)**

**INTRODUCTION**

**Ask to describe work/type of service**

- Type of service?
- Groups/clients that you work with?
- What is your role?
- What services/practices/initiatives do you offer?

**IMPACT ON CLIENT GROUP**

**What are the key issues facing your client group during the pandemic?**

- Have your clients been able to understand and adhere to the social distancing guidelines? (If yes - why/what has helped?/If no why not?)
- Have their experiences had any impact on being able to follow social distancing guidelines?
- Any existing physical or mental health problems
- Beliefs about consequences/ health beliefs
- Consequences for others/ self
- Needing to work/living arrangements, whether others are self-distancing in the same house/area
- Work/ Caring responsibilities, providing emotional support
- Peer pressure to socialise
- Managing to follow advice re facemasks indoors/ handwashing etc
- Government rules/punishments/messaging
- Feelings about losing normal life
- Change of routine/ habits
- What has been the impact of the pandemic on your clients being able to access services?
  - What has been the impact on any normal appointments for your group? (cancelled, delayed, unable to speak to appropriate support workers or health professionals)
- Have you noticed any impact on their physical/mental health? Could you describe some examples?
  - increased symptoms/side effects
  - Increase or change in presentation of conditions?
  - Impact on client ability to manage physical/mental health?
- Have there been any changes to the living arrangements of your clients? Could you describe some examples?
- Have your clients had any difficulties accessing daily provisions (including sanitation, hygiene products, food)? Could you describe some examples? Has anything helped them to access these provisions?

**CHALLENGES TO OPERATIONAL PRACTICES/SERVICE DELIVERY**

**What are the key challenges in delivering your service during the pandemic?**

- Ability to follow rules around social distancing and maintain delivery of service?
- Changes in services due to organisational or gov guidelines?
- Safety of staff/service users?
- Maintaining contact/relationships with service users?
- Impact on/emotional wellbeing of staff?
- Use of PPE and other measures to protect staff from the virus?

**Has your service made any adaptations in response to the challenges encountered during the pandemic?**

- - Any changes to current practices during lockdown to deal with issues?
  - New initiatives? (e.g. digital comms, buvidal, temp accommodation)
  - Different methods of communication?
  - Location of appointment? (e.g. online/telephone)
  - Any benefits to come out of changes from the pandemic?

**PROSEPECTION**

**Has the pandemic meant that you have any worries for the future (for your client group)?**

- Are there likely to be any lasting impacts/consequences of the pandemic on your clients?
- Are there likely to be any lasting impacts/consequences of the pandemic on service provision?

**How are these different from the worries you had before the pandemic?**

**Will this change the way you deliver your service in the future?**

- The way you connect with service users?
- How you support service users?
- How you operate daily
- The services/initiatives/practices that you deliver?
- How you support staff?

**Has this changed your priorities as a service for the future?**
